# The Responses of the Lipoxygenase Gene Family to Salt and Drought Stress in Foxtail Millet (*Setaria italica*)

**DOI:** 10.3390/life11111169

**Published:** 2021-11-02

**Authors:** Qianxiang Zhang, Yaofei Zhao, Jinli Zhang, Xukai Li, Fangfang Ma, Ming Duan, Bin Zhang, Hongying Li

**Affiliations:** 1 College of Agriculture, Shanxi Agricultural University, Jinzhong 030801, China; s20192116@stu.sxau.edu.cn (Q.Z.); s20192144@stu.sxau.edu.cn (J.Z.); 2 Shanxi Key Laboratory of Minor Crop Germplasm Innovation and Molecular Breeding, College of Agriculture, Shanxi Agricultural University, Jinzhong 030801, China; yfzhao@sxau.edu.cn (Y.Z.); xukai_li@sxau.edu.cn (X.L.); ffma@sxau.edu.cn (F.M.); mduan@sxau.edu.cn (M.D.)

**Keywords:** foxtail millet, lipoxygenase, gene family, abiotic stress, gene expression

## Abstract

Plant lipoxygenases (LOXs), a kind of non-heme iron-containing dioxygenases, participate plant physiological activities (especially in response to biotic and abiotic stresses) through oxidizing various lipids. However, there was few investigations on LOXs in foxtail millet (*Setaria italica*). In this study, we identified the *LOX* gene family in foxtail millet, and divided the total 12 members into three sub-families on the basis of their phylogenetic relationships. Under salt and drought stress, *LOX* genes showed different expression patterns. Among them, only *SiLOX7* showed up-regulated expression in Yugu1 (YG1) and Qinhuang2 (QH2), two stress-tolerant varieties, indicating that *SiLOX7* may play an important role in responses to abiotic stress. Our research provides a basis for further investigation of the role of *LOX* genes in the adaptation to abiotic stresses and other possible biological functions in foxtail millet.

## 1. Introduction

Drought and salt are both the main abiotic factors limiting the growth and development of plants, and thereby crop yield [1]. In many parts of the globe, the land has been suffering from salinization and increased drought condition, which will aggravate the negative impact on crops and endanger food production [2]. Drought and salt stress may cause membrane disorder, protein denaturation or inactivation by imposing osmotic stress on plants, and produce excessive reactive oxygen species leading to membrane oxidative damage, thereby causing plant growth disorders, photosynthesis inhibition and premature aging [3].

Lipoxygenases (oxygen oxidoreductase, EC 1.13.11.12; LOX) belong to non-heme iron-containing fatty dioxygenases which catalyze the degradation of polyunsaturated fatty acids [4,5]. This kind of iron-containing dioxygenase was widely present in fungi, animals, plants, algae, protozoa and bacteria cells [6,7]. The major substrates of LOX enzymes are linoleic acid and linolenic acid in plants [8]. Lipoxygenases catalyze the production of oxylipins, which play a key role during the whole growth period of plants, such as seed germination and plant growth, especially against biotic and abiotic stresses [9,10,11,12]. The down-regulation of lipoxygenase activity in rice seeds under storage conditions reduced the deterioration of seed quality. Additionally, rice seeds with reduced LOX enzyme activity showed higher germination efficiency after storage [13]. The metabolites of LOX pathway are the fatty acid oxidation products mediated by LOX, known as oxylipins, such as jasmonates (JA), green leaf volatiles (GLVs) and death acid (10-OPDA, 10-oxo-11-phytodienoic acid, and 10-OPEA, 10-oxo-11-phytoenoic acid, as well as a series of related 14- and 12-carbon metabolites), playing a major role in the responses to biotic and abiotic stress [14,15,16]. GLVs support plants for indirect defense by attracting the natural enemies of the herbivores, and can resist bacteria and fungi by having direct toxic effects on bacteria and fungi [17]. Death acids, a kind of products derived from 9-LOX in maize (*Zea mays*), were induced strongly when ingested by *Spodoptera exigua* to resist insects [18]. Among these three types of oxylipins, the 13-LOX derivative JA and its precursor (+)-12-oxo-phytodienoic acid (OPDA) play a major role in plant development and response to abiotic stress [19]. At present, there have been a number of reports proving the regulatory role of JA in plant drought and salt stress [20,21,22,23].

Plant LOXs are generally divided into two subfamilies, 9-LOXs and 13-LOXs according to their specific action positions of oxidized linoleic acids (LA) and linolenic acids (LnA) [24]. Furthermore, according to the structure and sequence similarity of LOXs, they are divided into two subfamilies, Type-Ⅰ and Type-Ⅱ. Type-Ⅰ LOXs have high sequence similarity (at least 75%) and their proteins lacks chloroplast transit peptides; Type-Ⅱ LOXs proteins have low sequence similarity (about 35%) and contain a chloroplast transit peptide. It is currently known that 9-LOXs subfamily belongs to the Type-Ⅰ, and 13-LOXs subfamily exists in the Type-Ⅰ and Type-Ⅱ [25]. Recently, it was found that 9-LOX was converted to 13-LOX by LOX-specific inhibitors. This location specificity may be related to the substitution of the canonical motif TV/R of 9-LOX in TdLOX2 with the unusual motif TV/K [26]. 

Up to now, new *LOX* gene families have been discovered continually to enrich plant LOXs [4,5,11,27,28,29]. Many of these *LOX* gene families exhibit different expression profiles, such as the 6 *LOX* genes in *Arabidopsis* and the 14 *LOX* genes in rice [5]. Among the above-identified LOX gene families, there are C_3_ photosynthesis Poaceae crops rice and wheat, and C_4_ photosynthesis Poaceae crops sorghum and maize. The responses of plant *LOX* genes to biotic and abiotic stresses were well reported. For instance, under hyperosmotic stress, durum wheat *TdLpx-A2* plays a key role in tolerance to abiotic stress response by inhibiting the overproduction of ROS [26]. In addition, some studies had detected quantitative trait locus related to the expression of different forms of LOX activity under a water deficit. The positive correlation between ChlMLOX activity and carotenoids may assume its involvement in nonphotochemical chlorophyll quenching, thereby protecting the photosynthetic structure from degradation by ROS [30]. In oriental melon, CmLOX10 significantly increases drought tolerance by mediating a JA biosynthesis pathway, and closing stomata to reduce water transpiration [16]. Expression pattern analysis also indicated that many *LOX* genes possibly be involved in metabolism and response to biotic and abiotic stress [31,32,33,34]. In addition, it has been found that LOX gene responds to temperature. In cotton, *GhLOX18* was only induced under cold stress, and more *GhLOX* genes showed altered expression in response to heat [33]. *LOX1*, *LOX3* and *LOX5* were identified in tomato that responded only to heat stress and *LOX11* to cold stress [35].

Foxtail millet (*Setaria italica* (L.) Beauv.), belonging to the *Panicoideae* subfamily and C4 crops, is an important regional food crop in arid and semi-arid areas. Due to its strong tolerance to drought and barren soil, foxtail millet plays an important role in food security in China and even East Asia [36,37]. Recently, foxtail millet has become a C_4_ model plant for exploring basic biological processes, with its short life cycle and small genome (about 430 Mb) [38]. 

In this study, we identified 12 *LOX* genes of foxtail millet and analyzed their distribution on chromosomes, encoded protein sequences, phylogenetic relationships, classification, conserved domains and expression patterns and physiological parameters under different abiotic stresses, which is helpful for investigating the molecular mechanism of stress tolerance in foxtail millet, as well as the related studies in other C_4_ crops.

## 2. Materials and Methods

### 2.1. Sequence Acquisition and Identification of Foxtail millet LOXs

To identify foxtail millet *LOX* gene family members, the sequences of foxtail millet were downloaded from Phytozome v12.1 database (http://phytozome.jgi.doe.gov/pz/portal.html; accessed on 1 October 2020) [39]. LOX sequences of *Arabidopsis thaliana* were accessed from TAIR website (https://Arabidopsis.org/; accessed on 1 October 2020) [40]. The LOX sequences of rice were downloaded from CHINA RICE DATA CENTER website (http://www.ricedata.cn/; accessed on 1 October 2020). Two main methods were used for the identification of the foxtail millet *LOX* gene family. First, with the BLAST program in TBtools [41], the Arabidopsis LOX protein sequences were used as queries to identify foxtail millet *LOX* genes. The superfluous sequences with the same chromosome locus were removed from the BLAST result. Second, we scanned of the proteome for the presence of “lipoxygenase” and “PLAT/LH2” domains. Then we excluded sequences that only had “lipoxygenase” or “PLAT/LH2”. All predicted protein sequences were identified.

The TBtools software was used to visualize the location of the acquired genes on the chromosomes [41]. The ExPASy bioinformatics website (https://www.expasy.org/; accessed on 1 October 2020) was used to predict the molecular weight and isoelectric point of the foxtail millet LOX proteins.

### 2.2. Multiple Sequence Alignment and Phylogenetic Tree Construction

The MUSCLE program [42], with default settings, was used to align the LOX protein sequences. Editing and visualization of alignment was on the Jalview [43].Then, six *Arabidopsis thaliana* LOX, twelve rice LOX and thirteen foxtail millet LOX proteins were used for building a phylogenetic tree. The phylogenetic tree was constructed with the Molecular Evolutionary Genetics Analysis (MEGA 7) software and the Neighbor-Joining method using 1000 bootstrap values [44]. Using the same methods generated another phylogenetic tree, only based on twelve foxtail millet LOX proteins.

### 2.3. Conserved Motifs, Protein Secondary Structure, and Subcellular Localizations Analyses

The MEME Suite (http://meme-suite.org/index.html; accessed on 1 October 2020) [45] was applied to search for motifs, and with default settings it was expected the maximum number to be found was set to 20. The TBtools [41] software was used to analyze the phylogenetic tree and motifs of foxtail millet LOX. In addition, sequence logos for the conserved LOX domain of Foxtail millet LOX proteins were constructed by WebLogo [46].

The protein secondary structure of the foxtail millet LOX proteins were predicted on SOPMA (https://npsa-prabi.ibcp.fr/cgi-bin/npsa_automat.pl?page=npsa_sopma.html; accessed on 1 October 2020). Subcellualr localizations of LOXs in foxtail millet were predicted by ProtComp9.0 (http://linux1.softberry.com/berry.phtml?group=programs&subgroup=proloc&topic=protcomppl; accessed on 1 October 2020). 

### 2.4. Plant Material and Stress Treatment

In this study, we used three varieties of foxtail millet with different sensitivities to salt and drought stress, ‘Qinhuang2′ (QH2, stress-tolerant), ‘Yugu1′ (YG1, stress-tolerant) and ‘AN04′ (stress-sensitive). Mature non-dormant seeds were sown into pots with nutrient soil and vermiculite (4:1, *v/v*). Plants were cultivated manual climatic box at Shanxi Agricultural University, and grown under controlled environmental setting of 28 ℃ day/22 ℃ night, 16 h light/8 h dark photoperiod, the light intensity was 14,000 lx. Seeds were sowed three rows per rack, one variety (20 plants) per row, four racks per treatment to ensure sufficient material. Stress treatment were performed when seedlings had six true leaves. For the drought stress treatments, when the water content of soil was less than 15%, start harvest samples. For the salt stress treatments, the seedlings were watered by 500 mmol/L NaCl solution. This certainly did not change the environment setting of manual climatic box. The above-ground part of a seedling was collected for physiological parameters determination and qRT-PCR. The materials at each time point were harvested three seedlings as biological replicates. All the samples were promptly frozen by liquid nitrogen after being harvested, and stored at −80 ℃ ultra-low temperature freezer to prevent RNA degradation.

### 2.5. Total RNA Extraction, Inverse Transcription, qRT-PCR Analysis

To extract total RNA, the frozen foxtail millet seedling samples were pestled with liquid nitrogen to powder. Total RNA from all simples were extracted with RNaiso Plus reagent (Takara Biotechnology, Beijing, China). To check the quality of the RNA samples, the ultra-low volume spectrometer (BioDrop, Cambridge, UK) was used to measure the concentration of RNA samples and A260/A280. Select RNA samples with A260/280 ratio between 1.8–2.1 for subsequent RNA reverse transcribed into cDNA. Used *PrimeScript*™ RT reagent Kit with gDNA Eraser (Takara Biotechnology, Beijing, China) and follow the manufacturer’s instructions to make RNA reverse transcribed into cDNA, and cleaned the gDNA. We diluted cDNA five times for qRT-PCR.

qRT-PCR was performed on a Bio-Rad CFX96 Real Time PCR machine, using TB Green^®^ *Premix Ex Taq*™ II (Takara Biotechnology, Dalian, China). qRT-PCR conditions: 95 ℃ for 30 s (initial denaturation), 95 ℃ for 5 s (denaturation),60 ℃ for 30 s (annealing and extending), step denaturation to annealing and extending for 40 cycles, 60 ℃ to 95 ℃ and increment 0.5 ℃ for 5 s (melt curve). Relative gene expression levels were calculated using the ∆∆Cq method [47] by Bio-Rad Manager 3.1 software, and *ACTIN* was used as an internal control. Gene-specific primers were designed using Primer Premier 5.0 software (Supplementary Table S1).

### 2.6. Determination of Physiological Parameters Related to Salt and Drought Stress

The activities of superoxide dismutase (SOD), peroxidase (POD) and concentration of malondialdehyde (MDA) were measured by spectrophotometer. Fresh seedling of 0.1 g were homogenized in 1.5 mL 0.1% trichloroacetic acid (TCA), and centrifuged at 12,000 rpm for 15 min at 4 °C; the resulting supernatant was used to measure MDA by 2-thiobarbituric acid (TBA) reaction [48]. We used 0.1 g of seedling samples homogenized in 1 mL pH 7.8, 50 mM phosphate buffer. Afterwards, the homogenate was centrifuged at 12,000 rpm for 15 min at 4 °C, and we used the supernatant to determine enzyme activities. SOD activity were measured using the nitrotetrazolium blue chloride Illumination method [49], and the activity of POD was measured in the light of the guaiacol method [50].

## 3. Results

### 3.1. Identification and Characterization of LOX Genes in Foxtail millet

To identify the *LOX* gene family in foxtail millet (*SiLOXs*), we used protein sequence homology searching and scanning of the proteome for the presence of “lipoxygenase” and “PLAT/LH2” domains. Firstly, we compared the protein sequences of Arabidopsis LOXs with the proteome of foxtail millet. As a result, 13 *SiLOXs* were identified and designated as *SiLOX1-13* (Supplemental Table S2). Through protein structure analysis, SiLOX12 was excluded, as it lacked the PLAT/LH2 domain. All the other 12 members were identified as *SiLOX* genes, with the standard encoding sequences of *LOX* genes [51]. 

**Figure 1:**
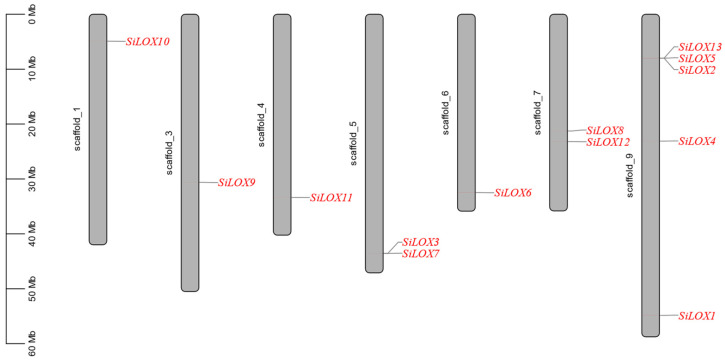
Distribution of *SiLOX* genes on foxtail millet chromosomes. The line on the gray strips shows the location of *SiLOX* genes on chromosomes. The scale in the left of picture is in megabases (Mb).

Foxtail millet *LOXs* were located on 7 chromosomes, except Chromosomes 2 and 8, with the most LOXs located on Chromosome 9. Among them, chromosome 9 had 5 *LOX* genes, chromosome 7 had 2 *LOX* genes, chromosome 1,3,4,6 only had one *LOX* gene (Figure 1). The CDS length of *SiLOX* genes varied between 1829–2970 bp, the encoded SiLOX proteins had 599 to 973 amino acids and the predicted molecular weight ranged from 69,190.23 Da to 106,479.19 Da. The isoelectric point (PI) ranged from 5.55 to 8.59 (Supplementary Table S2).

Using the GO entries in the Phytozome database, we searched different classification of GO function annotations of *LOX* gene in foxtail millet (Table 1). Among all *SiLOX* genes, *SiLOX1*, *SiLOX2*, *SiLOX3*, *SiLOX5, SiLOX7, SiLOX8, SiLOX9, SiLOX10* and *SiLOX13* all contained GO:0005515, GO:0016702, GO:0046872, GO:0055114. But *SiLOX4, SiLOX6 and SiLOX11* excluded GO:0005515. The GO items enriched in the *SiLOX* family were mainly concentrated in the molecular functions of oxidoreductase activity, metal ion binding, and the biological process of oxidation-reduction process. In addition, some *SiLOX* genes were annotated in molecular function of protein binding.

### 3.2. Phylogenetic Analysis of the SiLOX Gene Family

**Figure 2:**
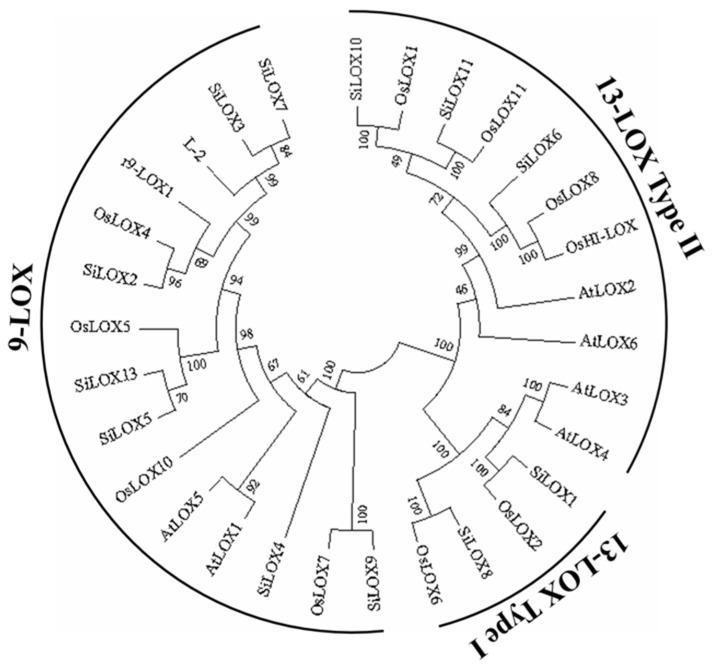
Neighbor-joining phylogenetic tree constructed with LOXs protein sequences from foxtail millet, *Arabidopsis thaliana* and rice. Accession numbers of sequences used to build the tree are as follows. Foxtail millet: SiLOX1, Seita.9G518800; SiLOX2, Seita.9G127800; SiLOX3, Seita.5G411600; SiLOX4, Seita.9G270500; SiLOX5, Seita.9G127700; SiLOX6, Seita.6G205300; SiLOX7, Seita.5G411700; SiLOX8, Seita.7G113700; SiLOX9, Seita.3G294500; SiLOX10, Seita.1G050700; SiLOX11, Seita.4G215400; SiLOX13, Seita.9G127600; *Arabidopsis thaliana*: AtLOX1, AT1G55020; AtLOX2, AT3G45140; AtLOX3, AT1G17420; AtLOX4, AT1G72520; AtLOX5, AT3G22400; AtLOX6, AT1G67560; rice: OsLOX1, LOC_Os02g10120; OsLOX2; LOC_Os03g08220; r9-LOX1, LOC_Os03g49260; OsLOX4, LOC_Os03g49350; OsLOX5, LOC_Os03g49380; L-2, LOC_Os03g52860; OsLOX6, LOC_Os04g37430; OsLOX7, LOC_Os05g23880; OsLOX8, LOC_Os08g39850; OsHI-LOX, LOC_Os08g39840; OsLOX10, LOC_Os11g36719; OsLOX11, LOC_Os12g37260.

To show the evolutionary relationships of the LOX family members in different species, we constructed a Neighbor-Joining phylogenetic tree with 1000 bootstrap of 31 plant LOX amino acid sequences including 12 SiLOXs, 12 OsLOXs (from rice) and 6 AtLOXs (from Arabidopsis). Phylogenetic tree categorizes the genes into 3 kinds of LOXs as 9-LOX, 13-LOX Type Ⅰ and 13-LOX Type Ⅱ (Figure 2). Among 12 SiLOXs, 8 SiLOXs (SiLOX2, SiLOX3, SiLOX4, SiLOX5, SiLOX7, SiLOX9 and SiLOX13) were characterized into 9-LOX enzymes group with 2 AtLOXs (AtLOX1 and AtLOX5) and 6 OsLOXs (L-2, r9-LOX1, OsLOX4, OsLOX5, OsLOX7 and OsLOX10). Furthermore, 5 SiLOXs (SiLOX1, SiLOX6, SiLOX8, SiLOX10 and SiLOX11) were characterized into 13-LOX with other AtLOXs and OsLOXs. Of the 5 13-LOXs in foxtail millet, 2 LOXs (SiLOX1 and SiLOX8) were grouped into type Ⅰ 13-LOX and only rice and foxtail millet LOXs were grouped into type I 13-LOX, similar to the results described previously [28]. Another 3 LOXs (SiLOX6, SiLOX10 and SiLOX11) were in type II 13-LOX. 9-LOXs were confirmed participating in growth, development and plant defense stress reactions [52,53], and 13-LOXs play an important role in jasmonic acid biosynthesis in Arabidopsis [54]. 

### 3.3. Conserved Protein Domain and Motifs of SiLOXs

**Figure 3:**
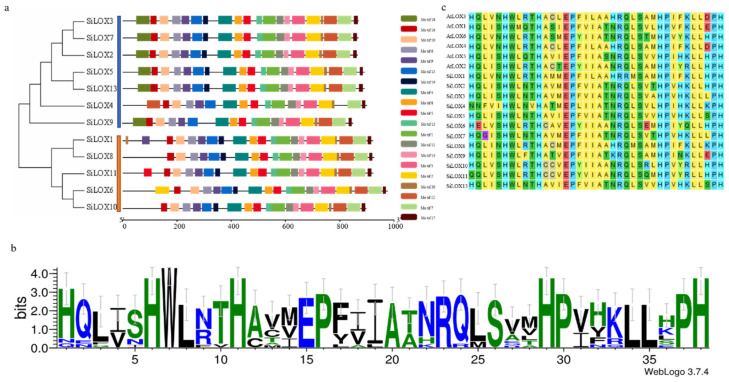
Phylogenetic tree, motifs, and conserved domains of SiLOXs. (**a**) Phylogenctic tree constructed by SiLOX protein sequences and motifs analysis; (**b**) Sequences logo of a 38-residue SiLOX motif in motif 1; (**c**) Alignment of a 38-residue conserved motif in AtLOX and SiLOX protein sequences.

PLAT/LH2(IPR001024) and lipoxygenase (IPR013819) domain were identified in almost all 12 SiLOX proteins (Table 2). To further understand the relationship among SiLOXs, we used 12 SiLOXs protein sequences for multiple alignment, and constructed a separate phylogenetic tree based on these multiple alignments. In addition, we used MEME Suite (Ver.5.3.3) to predict motifs in SiLOXs. SiLOXs were divided into 3 sub-families, as described above (9-LOX, 13-LOX Ⅰ and 13-LOX Ⅱ). We also found 20 motifs in SiLOXs, and nineteen of 20 motifs were shared among all SiLOXs protein (Figure 3a). Almost all 9-LOX members had all 20 motifs except SiLOX4 (which lacked 2 motifs). All 13-LOX missed motif 18. Among 20 motifs, motif 1 contained the LOX domain [His-(X)4-His-(X)4-His-(X)17-His-(X)8-His], consisted of 38 amino acids, almost conserved in all 12 SiLOXs. The first H of motif1 in SiLOX4 and SiLOX11 was replaced by N and Q (Figure 3b,c), All 12 SiLOX protein secondary structures were mainly composed of random coil and α-helix, with random coils in higher proportion. In addition, these secondary structures also contain extended strand and β-turn (Table 3).

For the prediction of the subcellular localization of the SiLOX proteins, SOPMA program was used. The results showed that 7 SiLOX proteins were located at cytoplasm (SiLOX1, SiLOX2, SiLOX3, SiLOX4, SiLOX7, SiLOX8 and SiLOX9) and 6 SiLOX proteins in chloroplast. As previously described, all 13-LOX type Ⅱ contained chloroplast transit peptide, whereas 13-LOX type Ⅰ might be localized in cytoplasm (Supplementary Table S2).

### 3.4. Physiological Responses of Different Varieties of Foxtail millet to Abiotic Stresses

**Figure 4:**
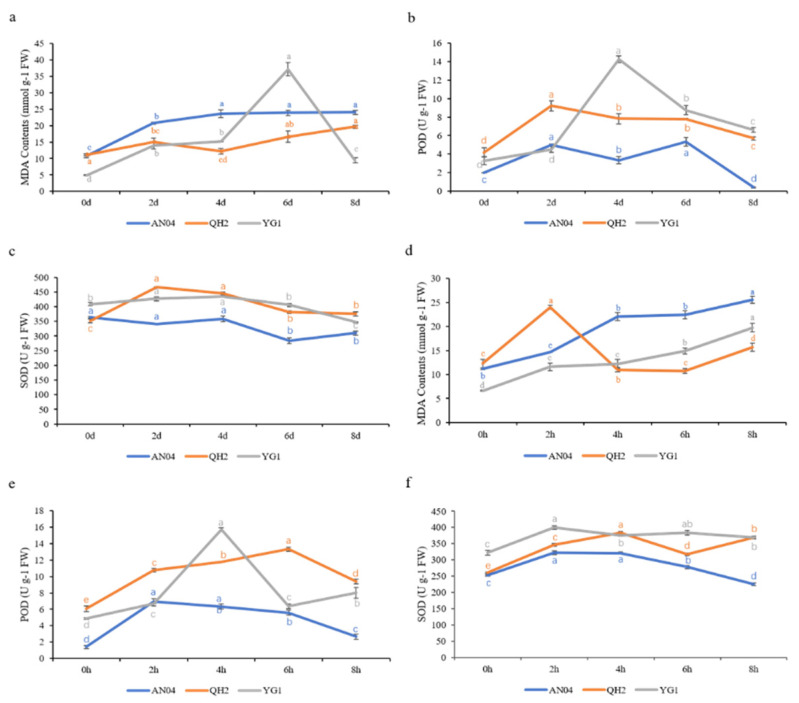
Physiological responses of AN04, YG1 and QH2 under drought and salt stress. (**a**–**c**) MDA content and activities of POD and SOD under drought stress; (**d**–**f**) Activities of POD, SOD and MDA content under salt stress. The lowercase letters above the line indicated the statistical significance at the level of 0.05 (*p* < 0.05).

We measured the physiological parameters related to stress in three varieties foxtail millet under two abiotic stresses, salinity and drought conditions. The results revealed that the trends of physiological indexes of the three varieties had an obvious relationship with their respective tolerance. Under drought stress, the MDA content of QH2 and YG1 was lower than that of AN04, while the MDA content of YG1 increased sharply from 4 to 6 days, then decreased to lower than that of AN04 from 6 to 8 days. Similarly, under salt stress, MDA content showed a similar variation trend, but the MDA content of QH2 increases from 0–2 d and is higher than that of AN04, and then began to decrease after 2 d and gradually falls below that of AN04. The content of MDA was a reflection of the peroxidation degree of the plant cell membrane, and was considered as one of the indicators of plant exposure to abiotic organisms. Compared with the tolerant varieties YG1 and QH2, the sensitive variety AN04 had a significantly higher MDA content and suffered more. Abiotic stress increases the content of ROS (mainly O^2−^ and H_2_O_2_) in plants, which can damage plant cells. Peroxidase (POD) and superoxide dismutase (SOD) were antioxidant enzymes in plants, which eliminate peroxidase damage by scavenging ROS such as H_2_O_2_ in plants. The results showed that the POD activity firstly increased and then decreased under salt stress and drought stress. As expected, POD activity of tolerant varieties YG1 and QH2 was higher than that of sensitive variety AN04. The POD activity of YG1 was significantly higher than that of QH2 at 4 d, 6 d (drought stress) and 4 h (salt stress). The activities of SOD and POD showed a similar trend under the two stress conditions, and the SOD activity in AN04 was lower than that in YG1 and QH2. Under drought stress, SOD activity of QH2 increased faster than that in YG1, and then decreased. In addition, SOD activity of QH2 was almost always lower than that in YG1 under salt stress (Figure 4).

### 3.5. Expression Analysis of SiLOX Genes under Salt and Drought Stress

**Figure 5:**
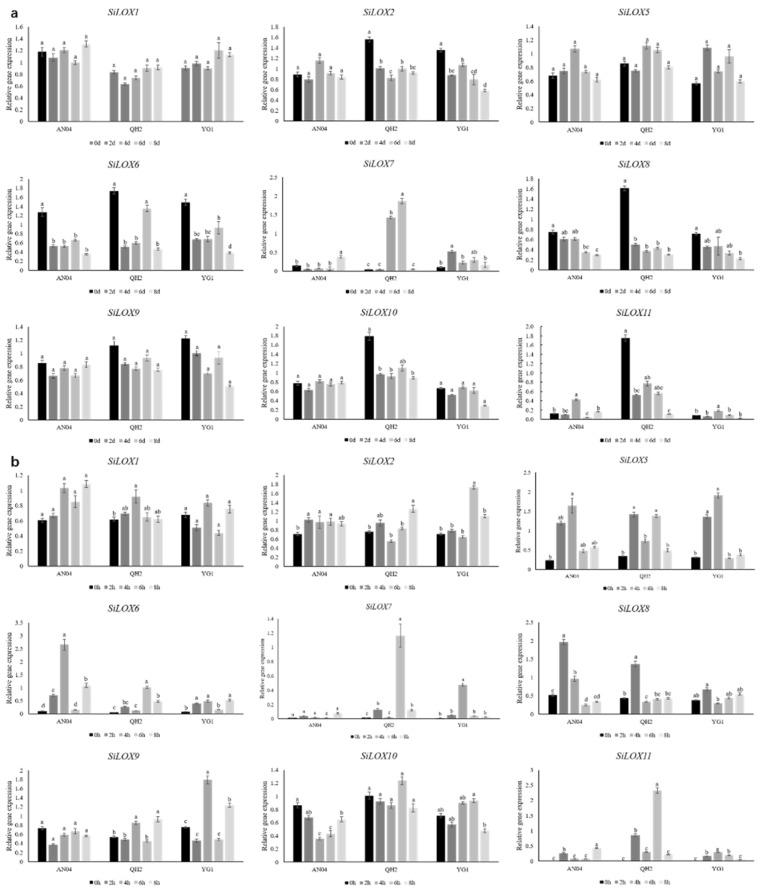
qRT-PCR analysis of LOX genes expression patterns in AN04, YG1 and QH2 under drought and salt stress. (**a**) Expression patterns of *SiLOX* genes under drought stress; (**b**) LOX gene expression pattern under salt stress. The lowercase letters above the histogram indicated the statistical significance at the level of 0.05 (*p* < 0.05).

To explore the potential roles of *LOX* genes in foxtail millet against abiotic stresses, we investigated the expressions of *SiLOX*s under salt and drought treatments. The results of qRT-PCR showed that the expressions of all detected *SiLOX* genes were different under each stress condition (Figure 5). To eliminate the influence of plant circadian rhythm and biological clock on gene expression levels during drought stress, leaves were sampled at 10 a.m. for every sampling points. For salt stress treatment during 12 h on the same day, we detected the gene expression level of untreated foxtail millet, and the expression pattern of SiLOXs were displayed by relative expression level which was a compared percentage of expression level of treated plants and control.

Under both salt and drought stress, these genes showed changed expression level. *SiLOX5* showed the tendency of significant increasing expression after salt and drought stress. *SiLOX1* in the three varieties showed high expression in all periods of drought and salt stress. *SiLOX2* showed a tendency to significantly reduced gene expression after drought stress, but increased under salt treatment. However, the expression of *SiLOX9* was significantly reduced under drought stress, with opposite pattern under salt stress. Under drought stress, the expression of *SiLOX8* and *SiLOX10* showed a downward trend. Under salt stress, the expression of *SiLOX8* increased first and then decreased, whereas the expression of *SiLOX10* decreased first and then increased.

In drought-tolerant varieties YG1 and QH2, the expression levels of *SiLOX7* were significantly upregulated from by salinity stress, and this increasing trend started at 2 h after treatment. The expression level of *SiLOX7* was increased sharply at 4 d and reach the maximum at 6 d in QH2 under drought treatment. However, in YG1, the expression level kept a high level after 2d , which was similar to the 9-LOX gene subfamily in cotton under drought and salt stress [33]. Interestingly, *SiLOX11* expression was considerably upregulated in both salt and drought stress only in QH2. In addition, *SiLOX6* was upregulated in drought-sensitive variety AN04. We analyzed the correlation between the expression of some LOX genes (SiLOX6, Si-LOX7 and SiLOX11) and physiological parameters under salt stress. The expression levels of these genes were significantly positively correlated with the content of POD, and SiLOX7 was the most significant. (Supplemantary Figure S1). These results indicated that *SiLOX7* and *SiLOX11* may regulate the responses to abiotic stress in foxtail millet. 

## 4. Discussion

With the development of genome sequencing technology, more and more *LOX* genes have been identified in different species. Moreover, research on the growth and development of the *LOX* gene family and its response to biological and abiotic stresses has gradually been extensive. However, relevant research in the past involved nothing about the foxtail millet *LOX* gene family. Almost all the plant *LOX* gene families that have been identified are based on the 6 members of the model plant Arabidopsis *LOX* gene family [54], such as 64 putative LOXs in 4 species of cotton [33], 14 deduced LOXs in tomato [11], 9 LOXs in sorghum [4], 13 LOXs in maize [29], 14 LOXs in rice [5] and more. In this research, we identified the *LOX* gene family in foxtail millet, which included 12 members more than twice as many members of the Arabidopsis *LOX* gene family, but one less than the members of the *LOX* gene family in rice. Interestingly, the *LOX* genes in millet and maize were also about twice as those in Arabidopsis. The differences in the number of members and their distribution on the chromosomes might indicate that the *LOX* gene had not been conserved in the evolutionary process. 

Through phylogenetic analysis, the foxtail millet *LOX* genes were classified into three subfamilies based on protein structure and sequence similarity. Consistent with the description of Feussner and Wasternack [51], the chloroplast transit peptide was present at the N-terminus of the Type-II LOX protein sequences and excluded from the Type-I LOX proteins. In addition, Type-Ⅱ LOX only contain 13-LOX Type Ⅱ, but 9-LOX and 13-LOX Type-I together constitute the Type-I LOX. Through the multiple sequence alignment of LOX protein sequences in foxtail millet, we observed some conserved motifs. Like tomato and cotton *LOX* genes, there was a highly conserved histidine-rich motif His-(X)4-His-(X)4-His-(X)17-His-(X)8-His in these conserved motifs, which contains 38 amino acids [11,33]. Interestingly, we found that SiLOX4 and SiLOX10 lacked the first His in this motif among 12 predicted SiLOXs. Therefore, we speculate that SiLOX4 and SiLOX10 may have different enzymatic activities from other members. This variation may be due to the diversity of LOX in the evolution process. Previous studies had shown that replacing the C-terminal isoleucine with valine can significantly increase the activity of lipoxygenase, but lipoxygenase may be inactivated due to the substitution of other amino acids. Among all the predicted amino acid sequences of SiLOX, 12 members have isoleucine at the C-terminus, while SiLOX4 had an asparagine substitution. Based on the above findings, SiLOX4 was more likely to have different enzyme activities from the other SiLOXs. According to the identification rules by Feussner and Wasternack, SiLOX12 was excluded from the *LOX* gene family, as it contained only the lipoxygenase domain [51].

LOX enzymes play key roles in almost all stages of plant life. In addition to participating in plant germination, growth, development and other biological activities, it also plays an important role in the response of plants to resist adversity [9,10,25]. The predicted *LOX* genes in foxtail millet are all annotated to the JA biosynthetic pathway. JA improves the tolerance of plants to salt and drought stress [20,21], suggesting that the members of the *LOX* gene family in foxtail millet might be related to improve the stress tolerance.

After analysis of the expression patterns of *SiLOX* genes under two abiotic stresses by qRT-PCR, we found that some of the genes show an up-regulation trend under drought or salt stress, which indicates that there may be similar tolerance mechanisms in foxtail millet to respond to drought and salt stress. In cotton, most *GhLOX* genes are related to heat and salt stress [33]. The similar expression pattern of *LOX* genes under different stresses may reflect the cross adaptation of plants. Some *SiLOX* genes showed different expression patterns under drought and salt stress. *SiLOX2*, *SiLOX6*, *SiLOX8* and *SiLOX9* were up-regulated under salt stress and down-regulated under drought stress. In QH2, *SiLOX10* and *SiLOX11* are down-regulated under drought stress and up-regulated under salt stress. It was noteworthy that *SiLOX7* in stress-tolerant varieties QH2 and YG1 showed a tendency of up-regulation under both stresses, especially in QH2. However, no significant changes were observed in sensitive variety AN04. The homologous genes of *SiLOX7* in Arabidopsis were *AtLOX1* and *AtLOX5*. Both the Arabidopsis mutants *lox1* and *lox5* have reduced stress tolerance with the increased MDA content [55]. Therefore, we speculated that *SiLOX7* plays an important role in improving the stress tolerance of foxtail millet.

By integrative analysis of the expression pattern of *SiLOX* genes, MDA content, the activities of SOD and POD both under drought and salt stress the possible mechanism of the influence of the *SiLOX* genes on the tolerance was explored in foxtail millet. In the three varieties of foxtail millet, the activities of SOD and POD all showed a trend of increasing at first then decreasing, whereas MDA generally rose slowly. It was observed from the results that the physiological parameters of the three varieties under stresses were correlated with the expression of *SiLOX7* to some extent. The activities of POD and SOD in YG1 and QH2 with high *SiLOX7* expression was significantly higher than those of AN04 with low *SiLOX7* expression, especially the activities of POD, which were more obvious (Figure S1). The MDA contents in AN04 were generally higher than those in QH2 and YG1. Plants produced ROS when suffered by abiotic stresses, which will damage the structure of plants in different aspects. Enzymes such as POD, SOD and some non-enzymatic antioxidants improved the stress tolerance of plants by scavenged ROS and free radicals produced by plants [56]. Therefore, the higher enzyme activities of POD and SOD in YG1 and QH2 than in AN04 may be related to their tolerance to stress. In addition, the content and increase scale of MDA in these two tolerant varieties were generally less than those in AN04, indicating that the antioxidant capacity of YG1 and QH2 was higher than that in AN04. We speculated that the trend of MDA in tolerant varieties was related to the increases in the activities of POD and SOD and the up-regulation of LOX genes such as *SiLOX7*, thereby reducing the degree of oxidation.

## 5. Conclusions

This research is the first genome-wide identification of the *LOX* gene family in foxtail millet. A total of 12 *SiLOX* genes were identified in foxtail millet, and they were highly conserved. The similarity and specificity of *SiLOX* genes in different varieties under drought and salt stress were found. Comprehensive analysis of gene expression patterns and physiological parameters related to stress suggested that *SiLOX7* could be involved in the stress tolerance in foxtail millet, such as affecting the activities of some antioxidant enzymes to resist the damage of peroxides to plants. The above research may support further analysis the function of foxtail millet *LOX* genes. 

## Figures and Tables

**Table 1:** Statistics list of GO classification of *LOX* gene in foxtail millet.

Gene ID	Gene Ontology
SiLOX1	GO:0005515; GO:0016702; GO:0046872; GO:0055114
SiLOX2	GO:0005515; GO:0016702; GO:0046872; GO:0055114
SiLOX3	GO:0005515; GO:0016702; GO:0046872; GO:0055114
SiLOX4	GO:0016702; GO:0046872; GO:0055114
SiLOX5	GO:0005515; GO:0016702; GO:0046872; GO:0055114
SiLOX6	GO:0016702; GO:0046872; GO:0055114
SiLOX7	GO:0005515; GO:0016702; GO:0046872; GO:0055114
SiLOX8	GO:0005515; GO:0016702; GO:0046872; GO:0055114
SiLOX9	GO:0005515; GO:0016702; GO:0046872; GO:0055114
SiLOX10	GO:0005515; GO:0016702; GO:0046872; GO:0055114
SiLOX11	GO:0016702; GO:0046872; GO:0055114
SiLOX13	GO:0005515; GO:0016702; GO:0046872; GO:0055114

**Table 2:** Identified protein domains in the lipoxygenase proteins in foxtail millet.

Name	PLAT/LH2 (IPR001024)	Lipoxygenase (IPR013819)
SiLOX1	81–221	232–900
SiLOX2	16–160	171–841
SiLOX3	15–161	172–844
SiLOX4	106–212	215–897
SiLOX5	21–166	177–863
SiLOX6	114–275	286–956
SiLOX7	19–163	174–843
SiLOX8	85–224	235–905
SiLOX9	19–148	159–824
SiLOX10	70–201	212–877
SiLOX11	83–211	222–898
SiLOX12		4–575
SiLOX13	21–166	177–863

**Table 3:** Secondary structure of lipoxygenase proteins in foxtail millet.

Protein	α-Helix	β-Turn	Extended Strand	Random Coil
SiLOX1	39.48%	5.45%	12.87%	42.20%
SiLOX2	37.15%	5.67%	13.66%	43.52%
SiLOX3	36.68%	5.88%	13.84%	43.60%
SiLOX4	35.56%	4.91%	13.49%	46.04%
SiLOX5	37.88%	5.52%	13.19%	43.40%
SiLOX6	35.66%	5.24%	14.29%	44.81%
SiLOX7	36.92%	5.09%	13.43%	44.56%
SiLOX8	39.37%	5.21%	13.02%	42.41%
SiLOX9	34.95%	5.90%	13.93%	45.22%
SiLOX10	38.48%	5.26%	13.98%	42.28%
SiLOX11	39.46%	4.46%	11.96%	44.13%
SiLOX13	36.75%	5.41%	13.87%	43.97%

## Data Availability

Not applicable.

## Supplementary Materials

The following are available online at https://www.mdpi.com/article/10.3390/life11111169/s1, Table S1: Primers information for qRT-PCR; Table S2: Information of the *SiLOX* genes. Including gene names and gene ID, chromosome location, gene length, CDS length, protein length, physical and chemical properties and prediction of subcellular location; Figure S1. The correlation between expression levels of *SiLOX* genes and physiological parameters under salt stress. (a) *SiLOX6*; (b) *SiLOX7*; (c) *SiLOX11*.
