# Supplementary material for: The Responses of the Lipoxygenase Gene Family to Salt and Drought Stress in Foxtail Millet (Setaria italica)
Source: Life (Basel). 2021 Nov 2;11(11):1169. doi: 10.3390/life11111169 (PMC8619181; doi:10.3390/life11111169)
Supplement: Supplementary file 1 [file life-11-01169-s001.zip › life-1419032-supplementary.pdf]

Article

The Responses of the Lipoxygenase Gene Family to Salt and Drought Stress in Foxtail Millet (*Setaria italica*)

Qianxiang Zhang, Yaofei Zhao, Jinli Zhang, Xukai Li, Fangfang Ma, Ming Duan, Bin Zhang and Hongying Li

Table S1. Primers information for qRT-PCR.

Gene	Forward primer Sequence (5'to3')	Reverse primer Sequence (5'to3')
ACTIN	TGC TCA GTG GAG GCT CAA CA	CAA GAC ACT GTA CTT GCG CTC
SiLOX1	AAA TCA CTG GCT GAG GAC ACA T	CAG GAG CTT GAA GAT CGG GT
SiLOX2	GGT CCT CGG AAA TGT GTT GG	GCT GAA CTT TAC GCA GGC TTA
SiLOX5	GGA CCT AAG GCA GTA TGG A	AGG ATG AAG AGC TTG TTG TT
SiLOX6	CTG CTG TCC TCG CAC TCC	CTC GCT GTC ATC GTT CCA T
SiLOX7	GAC CGT CTT CCC TCG CAA	GTC GTC GGG GTA GTA GAT GG
SiLOX8	CCC AAC AGC GTC ACC ATC TA	CCC CGC CCG AGT ATA ATG AG
SiLOX9	GCG GGT GAT GAT GAC ATA AGT A	TCT TTG TGG CTA TGA TGA ACG
SiLOX10	TAC CAC TAC GGC GGC TAC TT	TCT GCG TTG GGA GCA TGT C
SiLOX11	AAC CTC CTG TCG TCG CAC TC	GGG GTC CTT GTT CCT ACT ATC G

Table S2. Information of the *SiLOX* genes. Including gene names and gene ID, chromosome location, gene length, CDS length, protein length, physical and chemical properties and prediction of subcellular location.

Name	Gene ID	Location coordinates	Genomic	CDS	Protein length	Molecule weight	PI	Subcellular localization	Signal peptide
SiLOX1	Seita.9G518800	54841933-54848489	6556	2799	917	98855.37	7.43	Cytoplasmic	
SiLOX2	Seita.9G127800	8006339-8010980	4641	2638	864	96694.66	5.55	Cytoplasmic	
SiLOX3	Seita.5G411600	43553995-43557386	3391	2647	867	97245.64	6.14	Cytoplasmic	
SiLOX4	Seita.9G270500	23099870-23104420	4550	2738	897	98855.37	8.59	Cytoplasmic	
SiLOX5	Seita.9G127700	7994348-8000356	6008	2708	887	100527.00	5.99	Chloroplast	Chloroplast Transit peptide
SiLOX6	Seita.6G205300	32480283-32487466	7183	2970	973	106479.19	7.1	Chloroplast	Chloroplast Transit peptide
SiLOX7	Seita.5G411700	43562736-43566125	3389	2638	864	97095.12	5.93	Cytoplasmic	
SiLOX8	Seita.7G113700	21259335-21263951	4616	2815	922	102961.99	6.29	Cytoplasmic	
SiLOX9	Seita.3G294500	30615401-30619250	3849	2586	847	96169.80	8.35	Cytoplasmic	
SiLOX10	Seita.1G050700	4897008-4900939	3931	2729	894	100780.50	5.94	Chloroplast	Chloroplast Transit peptide
SiLOX11	Seita.4G215400	33369525-33372960	3435	2809	920	103881.63	5.84	Chloroplast	Chloroplast Transit peptide
SiLOX12	Seita.7G142300	23189645-23192844	3199	1829	599	69190.23	5.99	Chloroplast	Chloroplast Transit peptide
SiLOX13	Seita.9G127600	8006339-8010980	4641	2638	887	100857.10	8.1	Chloroplast	Chloroplast Transit peptide

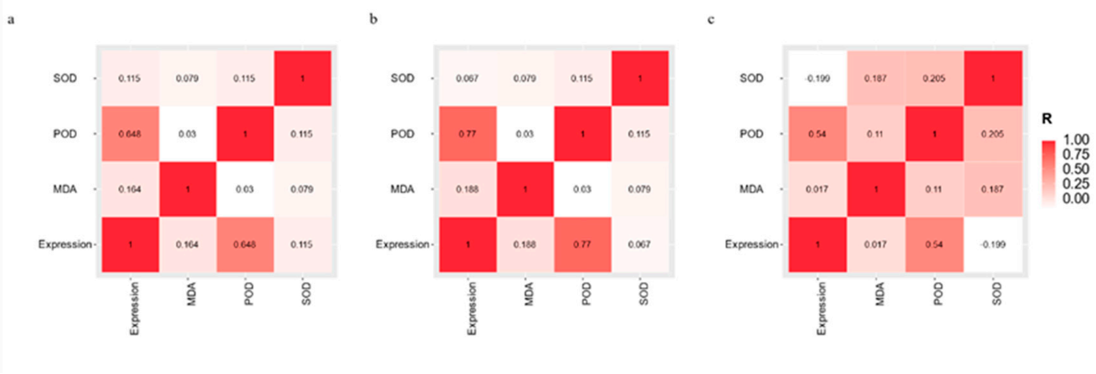

Figure S1. The correlation between expression levels of *SiLOX* genes and physiological parameters under salt stress. (a) *SiLOX6*; (b) *SiLOX7*; (c) *SiLOX11*.
